# Insulin secretagogue use and circulating inflammatory C–C chemokine levels in breast cancer patients

**DOI:** 10.1016/j.dib.2017.02.031

**Published:** 2017-02-16

**Authors:** Zachary A.P. Wintrob, Jeffrey P. Hammel, George K. Nimako, Zahra S. Fayazi, Dan P. Gaile, Alan Forrest, Alice C. Ceacareanu

**Affiliations:** a State University of New York at Buffalo, Dept. of Pharmacy Practice, NYS Center of Excellence in Bioinformatics and Life Sciences, 701 Ellicott Street, Buffalo, NY 14203, USA; b Cleveland Clinic, Dept. of Biostatistics and Epidemiology, 9500 Euclid Ave., Cleveland, OH 44195, USA; c State University of New York at Buffalo, Dept. of Biostatistics, 718 Kimball Tower, Buffalo, NY 14214, USA; d The UNC Eshelman School of Pharmacy, Division of Pharmacotherapy and Experimental Therapeutics, Campus Box 7569, Chapel Hill, NC 27599, USA; e Roswell Park Cancer Institute, Dept. of Pharmacy Services, Elm & Carlton Streets, Buffalo, NY 14263, USA

**Keywords:** Inflammation, Inflammatory cytokines, Secretagogue, C–C chemokines, CCL-2, CCL-3, CCL-4, CCL-5, Breast cancer, Diabetes, Monocyte infiltration, Activated macrophage, Cancer outcomes, Cancer prognosis

## Abstract

Monocytes’ infiltration into the tumor tissue and their activation to tumor-associated macrophages is an essential step in tumor development, also playing a critical role in an eventual metastasis. Stimulation of endogenous insulin production by oral insulin secretagogue treatment has the potential to interfere with the production and release of C–C chemokines, a group of potent inflammatory cytokines acting as monocyte chemo-attractants and influencing their behavior in the tumor microenvironment.

Studied plasma samples were collected under a previously reported study design involving a population of women diagnosed with breast cancer presenting with or without type 2 diabetes mellitus at the time of breast cancer diagnosis (Wintrob et al., 2017, 2016) [1,2]. The data presented here shows the relationship between pre-existing use of insulin secretagogue, the inflammatory C–C chemokine profiles at the time of breast cancer diagnosis, and subsequent cancer outcomes. A Pearson correlation analysis stratified by secretagogue use and controls was implemented to evaluate the relationship between the investigated biomarkers and respectively each of these biomarkers and the other relevant reported cytokine datasets derived from the same patient population (Wintrob et al., 2017, 2016) [1,2].

**Specifications table**

**Table:** 

Subject area	Clinical and Translational Research
More specific subject area	Biomarker Research, Cancer Epidemiology
Type of data	Tables
How data was acquired	The TYBRES study was designed to assess the relationship between utilization of specific diabetes mellitus pharmacotherapies, breast cancer outcomes, and biomarker profiles, of which the associations between medication use and adipokines’ circulation have been recently reported [1], [2]. The data presented here was obtained by linking new biomarker profiles to the original TYBRES patient database. Tumor registry query was followed by vital status ascertainment, and medical records review as described [1], [2]. Luminex®-based quantitation from plasma samples was conducted for the following inflammatory C–C chemokine ligands: Chemokine ligand 2, CCL-2 (monocyte chemoattractant protein 1, MCP-1); chemokine ligand 3, CCL-3 (macrophage inflammatory protein 1α, MIP-1α); chemokine ligand 4, CCL-4 (macrophage inflammatory protein 1β, MIP-1β); and chemokine ligand 5, CCL-5 (regulated on activation normal T cell expressed and secreted, RANTES). A Luminex®200™ instrument with Xponent 3.1 software was used to acquire all data
Data format	Analyzed
Experimental factors	Above described biomarkers were determined from the corresponding plasma samples collected at the time of breast cancer diagnosis.
Experimental features	This dataset included 97 adult female cases diagnosed with type 2 diabetes and incident breast cancer and 194 matched controls with newly diagnosed breast cancer, but no diabetes diagnosis. Clinical and treatment history were evaluated in relationship with cancer outcomes and C-C chemokine profiles. A correlation analysis was performed.
Data source location	United States, Buffalo, NY – 42° 53′ 50.3592″N; 78° 52′ 2.658″W
Data accessibility	The data is with this article

**Value of the data**

• Monocytes’ mobilization to the tumor location is a chemotactic response mediated by pro-inflammatory C–C chemokine ligands: CCL-2, CCL-3, CCL-4, and CCL-5 [3]. Their combined contribution determines specific tumor environment changes many of which are responsible for metastasis.
• CCL-2 was the first described tumor-derived factor while later has been found to also be elevated among type 2 diabetes patients [4], [5]. CCL-2 promotes tumor metastasis through secretion of CCL-3. Given its crucial role, CCl-2 is currently explored as a diagnostic and prognostic biomarker [6], [7], [8], [9]. CCL-4 and CCL-5 are reported to facilitate metastasis and contribute to disease progression [10], [11], [12]. CCL-5 is currently considered as a therapeutic target for breast cancer [13].
• Present data shows the observed relationship between history of insulin secretagogue use, circulating C–C chemokines at breast cancer diagnosis and cancer outcomes.
• This data provides additional detail for the design of future studies investigating the relationship between insulin production and inflammation leading to breast cancer metastasis.
• Our observations have the potential to guide research investigating the use of C–C chemokines as diagnostic and/or prognostic indicators.

## 1 Data

Reported data represents the observed association between use of insulin secretagogues preceding breast cancer and the inflammatory C–C chemokine profiles at the time of cancer diagnosis in women with diabetes mellitus (Table 1). Data in Table 2 includes the observed correlations between the measured biomarkers stratified by type 2 diabetes mellitus pharmacotherapy and controls, as well as correlations with other inflammatory adipokines reported by us in the past: tumor necrosis factor α, interleukin 1β and its receptor antagonist, and interleukin 6. The details regarding tumor necrosis factor α, interleukin 1β and its receptor antagonist, and interleukin 6 determination from plasma, their association with cancer outcomes and use of insulin secretagogues has been previously reported [1], [2].

## 2 Experimental design, materials and methods

Evaluation of pro-inflammatory cytokine profiles association with insulin secretagogue use and BC outcomes was carried out under two protocols approved by both Roswell Park Cancer Institute (EDR154409 and NHR009010) and the State University of New York at Buffalo (PHP0840409E). Demographic and clinical patient information was linked with cancer outcomes and pro-inflammatory cytokine profiles of corresponding plasma specimen harvested at BC diagnosis and banked in the Roswell Park Cancer Institute Data Bank and Bio-Repository.

### 2.1 Study population

All incident breast cancer cases diagnosed at Roswell Park Cancer Institute (01/01/2003–12/31/2009) were considered for inclusion (*n*=2194). Medical and pharmacotherapy history were used to determine the baseline presence of diabetes [1], [2].

### 2.2 Inclusion and exclusion criteria

All adult women with pre-existing diabetes at breast cancer diagnosis having available banked treatment-naïve plasma specimens (blood collected prior to initiation of any cancer-related therapy – surgery, radiation or pharmacotherapy) in the Institute׳s Data Bank and Bio-Repository were included.

Subjects were excluded if they had prior cancer history or unclear date of diagnosis, incomplete clinical records, type 1 or unclear diabetes status. For a specific breakdown of excluded subjects, please see the original research article by Wintrob et al. [1].

A total of 97 female subjects with breast cancer and baseline diabetes mellitus were eligible for inclusion in this analysis.

### 2.3 Control-matching approach

Each of the 97 adult female subjects with breast cancer and diabetes mellitus (defined as “cases”) was matched with two other female subjects diagnosed with breast cancer, but without baseline diabetes mellitus (defined as “controls”). The following matching criteria were used: age at diagnosis, body mass index category, ethnicity, menopausal status and tumor stage (as per the American Joint Committee on Cancer). Some matching limitations applied [1].

### 2.4 Demographic and clinical data collection

Clinical and treatment history was documented as previously described [1]. Vital status was obtained from the Institute׳s Tumor Registry, a database updated biannually with data obtained from the National Comprehensive Cancer Networks’ Oncology Outcomes Database. Outcomes of interest were breast cancer recurrence and/or death. The specific treatment groups have been defined according to the mechanism of action of their respective diabetes pharmacotherapy. Receiving any of the following pharmacotherapies alone or in combination: sulfonylureas (glimepiride, glipizide, and glyburide), meglitinides (nateglinide, repaglinide), alpha-glucosidase inhibitors (acarbose, miglitol), glucagon-like peptide-1 receptor agonists (exenatide, liraglutide), led to assigning the subject to the “any secretagogue” user group, whereas the “no secretagogue” user group included patients receiving one or more of the following treatment options: biguanides (metformin) and thiazolidinediones (pioglitazone, rosiglitazone) or no oral pharmacotherapy [1]. Of note is that each of the two groups, *any secretagogue* and *no secretagogue*, included 11 and respectively 9 insulin users.

### 2.5 Plasma specimen storage and retrieval

All the plasma specimens retrieved from long-term storage were individually aliquoted in color coded vials labeled with unique, subject specific barcodes. Overall duration of freezing time was accounted for all matched controls ensuring that the case and matched control specimens had similar overall storage conditions. Only two instances of freeze-thaw were allowed between biobank retrieval and biomarker analyses: aliquoting procedure step and actual assay.

### 2.6 Luminex® assays

A total of 5 biomarkers – chemokine ligand 2, CCL-2 (monocyte chemoattractant protein 1, MCP-1); chemokine ligand 3, CCL-3 (macrophage inflammatory protein 1α, MIP-1α); chemokine ligand 4, CCL-4 (macrophage inflammatory protein 1β, MIP-1β); and chemokine ligand 5, CCL-5 (regulated on activation normal T cell expressed and secreted, RANTES) – were quantified according to the manufacturer protocol. The HCYTOMAG-60K Luminex® biomarker panel (Millipore Corporation, Billerica, MA) was utilized in this study. Tumor necrosis factor α, interleukin 1β, interleukin 1β receptor antagonist, interleukin 6, and interleukin 10 determinations were done according to the manufacturer protocol as previously reported [1], [2].

### 2.7 Biomarker-pharmacotherapy association analysis

Biomarker cut-point optimization was performed for each analyzed biomarker. Biomarker levels constituted the continuous independent variable that was subdivided into two groups that optimized the log rank test among all possible cut-point selections yielding a minimum of 10 patients in any resulting group. Quartiles were also constructed. The resultant biomarker categories were then tested for association with type 2 diabetes mellitus therapy and controls by Fisher׳s exact test. The continuous biomarker levels were also tested for association with diabetes therapy and controls across groups by the Kruskal–Wallis test and pairwise by the Wilcoxon rank sum. Multivariate adjustments were performed accounting for age, tumor stage, body mass index, estrogen receptor status, and cumulative comorbidity. The biomarker analysis was performed using R Version 2.15.3. Please see the original article for an illustration of the analysis workflow [1].

Correlations between biomarkers stratified by type 2 diabetes mellitus pharmacotherapy and controls were assessed by the Pearson method. Correlation models were constructed both with and without adjustment for age, body mass index, and the combined comorbidity index. Correlation analyses were performed using SAS Version 9.4.

## Funding sources

This research was funded by the following grant awards: Wadsworth Foundation Peter Rowley Breast Cancer Grant awarded to A.C.C. (UB Grant Number 55705, Contract CO26588).

## Figures and Tables

**Table 1:** Pro-inflammatory Cytokine Associations with Secretagogue Use.

Biomarker	Biomarker grouping	Concentration	Control	No secretagogue	Any secretagogue	Unadjusted *p*-value (MVP)			
						p^1^	p^2^	p^3^	Global test
CCL-2 (MCP-1, pg/ml)	Median (25th–75th)	–	304 (221–392)	296 (252–382)	301 (216–391)	0.810 (0.610)	1.000 (0.520)	0.900 (0.330)	0.970 (0.710)
	Quartiles	1.6 to 225.6	52 (26.9%)	8 (17.0%)	13 (26.0%)	0.180	0.900	0.620	0.540
		227.7 to 302.5	42 (21.8%)	17 (36.2%)	13 (26.0%)				
		303.7 to 388.6	50 (25.9%)	11 (23.4%)	11 (22.0%)				
		391.9 to 4531.2	49 (25.4%)	11 (23.4%)	13 (26.0%)				
	OS-Based Optimization	**1.6 to 395.8**⁎	146 (75.6%)	36 (76.6%)	38 (76.0%)	0.890 (0.610)	0.960 (0.550)	0.950 (0.880)	0.990 (0.850)
		398.5 to 4531.2	47 (24.4%)	11 (23.4%)	12 (24.0%)				
	DFS-Based Optimization	1.6 to 170.4	22 (11.4%)	3 (6.4%)	6 (12.0%)	0.430 (0.100)	0.910 (0.480)	0.490 (0.390)	0.580 (0.330)
		**172.4 to 4531.2**	171 (88.6%)	44 (93.6%)	44 (88.0%)				
									
CCL-3 (MIP-1α, ng/ml)	Median (25th–75th)	–	3.82 (2.38–6.95)	5.63 (3.18–10.09)	3.86 (1.97–9.11)	0.051 (0.160)	0.760 (0.880)	0.230 (0.320)	0.160 (0.290)
	Quartiles	0.36 to 2.37	49 (25.3%)	9 (19.1%)	15 (30.0%)	0.160	0.360	0.540	0.280
		2.41 to 4.02	53 (27.3%)	9 (19.1%)	11 (22.0%)				
		4.07 to 7.96	51 (26.3%)	12 (25.5%)	9 (18.0%)				
		8.11 to 390.27	41 (21.1%)	17 (36.2%)	15 (30.0%)				
	OS-Based Optimization	0.36 to 4.02	102 (52.6%)	18 (38.3%)	26 (52.0%)	0.080 (0.080)	0.940 (0.450)	0.180 (0.350)	0.210 (0.180)
		**4.07 to 390.27**⁎	92 (47.4%)	29 (61.7%)	24 (48.0%)				
	DFS-Based Optimization	0.36 to 4.02	102 (52.6%)	18 (38.3%)	26 (52.0%)	0.080 (0.080)	0.940 (0.450)	0.180 (0.350)	0.210 (0.180)
		**4.07 to 390.27**	92 (47.4%)	29 (61.7%)	24 (48.0%)				
									
CCL-4 (MIP-1β, pg/ml)	Median (25th–75th)	–	23.00 (16.54–32.87)	28.74 (20.74–44.77)	27.48 (20.20–37.74)	0.009 (0.009)	0.060 (0.250)	0.380 (0.380)	0.012 (0.019)
	Quartiles	1.60 to 17.56	56 (28.9%)	8 (17.0%)	9 (18.0%)	0.220	0.370	0.950	0.370
		17.58 to 23.77	48 (24.7%)	11 (23.4%)	14 (28.0%)				
		23.92 to 34.81	48 (24.7%)	12 (25.5%)	12 (24.0%)				
		34.94 to 660.94	42 (21.6%)	16 (34.0%)	15 (30.0%)				
	OS-Based Optimization	1.60 to 12.40	18 (9.3%)	3 (6.4%)	3 (6.0%)	0.770 (0.370)	0.580 (0.230)	1.000 (0.870)	0.770 (0.460)
		**12.58 to 660.94**	176 (90.7%)	44 (93.6%)	47 (94.0%)				
	DFS-Based Optimization	1.60 to 13.59	26 (13.4%)	4 (8.5%)	3 (6.0%)	0.370 (0.390)	0.160 (0.190)	0.710 (0.810)	0.270 (0.360)
		**13.69 to 660.94**	168 (86.6%)	43 (91.5%)	47 (94.0%)				
									
CCL-5 (RANTES, pg/ml)	Median (25th–75th)	–	7158 (3460–14543)	5802 (4168–10391)	5673 (3269–8904)	0.640 (0.810)	0.090 (0.240)	0.300 (0.220)	0.230 (0.400)
	Quartiles	0 to 3446	49 (25.3%)	8 (17.0%)	16 (32.0%)	0.051	0.330	0.350	0.110
		3500 to 6307	41 (21.1%)	18 (38.3%)	14 (28.0%)				
		6381 to 13442	48 (24.7%)	13 (27.7%)	11 (22.0%)				
		13442 to 57898	56 (28.9%)	8 (17.0%)	9 (18.0%)				
	OS-Based Optimization	0 to 3183	42 (21.6%)	8 (17.0%)	11 (22.0%)	0.480 (0.650)	0.960 (0.810)	0.540 (0.770)	0.770 (0.860)
		**3212 to 57898**⁎	152 (78.4%)	39 (83.0%)	39 (78.0%)				
	DFS-Based Optimization	0 to 16821	160 (82.5%)	43 (91.5%)	46 (92.0%)	0.140 (0.029)	0.110 (0.160)	1.000 (0.910)	0.100 (0.080)
		**16982 to 57898**	34 (17.5%)	4 (8.5%)	4 (8.0%)				

⁎ Overall survival (OS)- and disease-free survival (DFS)-optimized biomarker ranges associated with poorer outcomes are represented in bold. Unadjusted *p*-values: p1, compares *no insulin versus control*; p2, compares *any insulin versus control*; p3, compares *any insulin versus no insulin* (as per Kruskal–Wallis test); global test, compares *all categories* (as per Wilcoxon, type 3 error test); MVP, denotes the *p*-value of each multivariate adjusted analysis corresponding to the earlier described unadjusted analyses. For more information, please see Section 2.7 below and our previously published analysis work flow [1]. MVP=*p*-value of the multivariate adjusted analysis. Chemokine ligand 2, CCL-2 (monocyte chemoattractant protein 1, MCP-1); chemokine ligand 3, CCL-3 (macrophage inflammatory protein 1α, MIP-1α); chemokine ligand 4, CCL-4 (macrophage inflammatory protein 1β, MIP-1β); chemokine ligand 5, CCL-5 (regulated on activation normal T cell expressed and secreted, RANTES).

**Table 2:** Pro-inflammatory Cytokine Correlations by Secretagogue Use.

Compared biomarkers		Group	Unadjusted correlation				Adjusted correlation		
			Pearson correlation	95% confidence interval	*p*-Value		Pearson correlation	95% confidence interval	*p*-Value
CCL-2 (MCP-1)	CCL-3 (MIP-1α)	All subjects (*n*=291)	−0.042	−0.156 to 0.074	0.480		−0.043	−0.158 to 0.073	0.463
		Controls (*n*=194)	−0.034	−0.174 to 0.108	0.636		−0.029	−0.170 to 0.114	0.695
		No secretagogue (*n*=43)	−0.091	−0.381 to 0.215	0.560		−0.125	−0.420 to 0.194	0.440
		Any secretagogue (*n*=54)	−0.162	−0.412 to 0.110	0.238		−0.158	−0.416 to 0.122	0.263
								
CCL-2 (MCP-1)	CCL-4 (MIP-1β)	All subjects (*n*=291)	0.008	−0.107 to 0.123	0.897		0.008	−0.108 to 0.123	0.892
		Controls (*n*=194)	−0.002	−0.143 to 0.139	0.974		−0.001	−0.143 to 0.141	0.990
		No secretagogue (*n*=43)	0.057	−0.248 to 0.351	0.716		0.048	−0.268 to 0.354	0.768
		Any secretagogue (*n*=54)	0.078	−0.194 to 0.339	0.574		0.082	−0.198 to 0.350	0.564
								
CCL-2 (MCP-1)	CCL-5 (RANTES)	All subjects (*n*=291)	**−0.172**	**−0.281 to −0.058**	**0.003**		**−0.174**	**−0.283 to −0.059**	**0.003**
		Controls (*n*=194)	**−0.257**	**−0.384 to −0.121**	**<0.001**		**−0.251**	**−0.379 to −0.113**	**<0.001**
		No secretagogue (*n*=43)	**0.416**	**0.132 to 0.637**	**0.005**		**0.422**	**0.127 to 0.648**	**0.006**
		Any secretagogue (*n*=54)	−0.158	−0.409 to 0.114	0.249		−0.183	−0.436 to 0.098	0.196
								
CCL-2 (MCP-1)	IL-1β	All subjects (*n*=291)	−0.037	−0.151 to 0.078	0.529		−0.036	−0.151 to 0.080	0.545
		Controls (*n*=194)	−0.008	−0.148 to 0.133	0.916		−0.016	−0.158 to 0.126	0.821
		No secretagogue (*n*=43)	−0.051	−0.346 to 0.253	0.744		−0.062	−0.366 to 0.255	0.703
		Any secretagogue (*n*=54)	−0.104	−0.362 to 0.168	0.450		−0.067	−0.336 to 0.213	0.639
								
CCL-2 (MCP-1)	IL-1Ra	All subjects (*n*=291)	−0.014	−0.129 to 0.101	0.815		−0.011	−0.127 to 0.104	0.849
		Controls (*n*=194)	−0.007	−0.148 to 0.134	0.923		−0.004	−0.146 to 0.138	0.953
		No secretagogue (*n*=43)	−0.023	−0.321 to 0.280	0.885		−0.032	−0.340 to 0.282	0.844
		Any secretagogue (*n*=54)	0.092	−0.180 to 0.351	0.507		0.075	−0.205 to 0.343	0.600
								
CCL-2 (MCP-1)	TNF-α	All subjects (*n*=291)	−0.013	−0.128 to 0.102	0.824		−0.008	−0.123 to 0.108	0.899
		Controls (*n*=194)	−0.001	−0.142 to 0.140	0.987		−0.018	−0.159 to 0.125	0.808
		No secretagogue (*n*=43)	−0.055	−0.350 to 0.249	0.722		−0.040	−0.347 to 0.275	0.805
		Any secretagogue (*n*=54)	0.127	−0.146 to 0.382	0.357		0.155	−0.126 to 0.413	0.273
								
CCL-2 (MCP-1)	IL-6	All subjects (*n*=291)	0.010	−0.105 to 0.124	0.870		0.007	−0.109 to 0.122	0.910
		Controls (*n*=194)	0.015	−0.126 to 0.156	0.831		0.016	−0.126 to 0.158	0.825
		No secretagogue (*n*=43)	0.002	−0.298 to 0.303	0.987		0.005	−0.307 to 0.316	0.975
		Any secretagogue (*n*=54)	−0.165	−0.414 to 0.108	0.230		−0.122	−0.385 to 0.159	0.392
								
CCL-3 (MIP-1α)	CCL-4 (MIP-1β)	All subjects (*n*=291)	**0.267**	**0.157 to 0.371**	**<0.001**		**0.268**	**0.157 to 0.372**	**<0.001**
		Controls (*n*=194)	**0.239**	**0.102 to 0.368**	**<0.001**		**0.235**	**0.097 to 0.365**	**0.001**
		No secretagogue (*n*=43)	**0.551**	**0.301 to 0.731**	**<0.001**		**0.581**	**0.329 to 0.756**	**<0.001**
		Any secretagogue (*n*=54)	**0.750**	**0.603 to 0.847**	**<0.001**		**0.737**	**0.580 to 0.842**	**<0.001**
								
CCL-3 (MIP-1α)	CCL-5 (RANTES)	All subjects (*n*=291)	0.091	−0.025 to 0.204	0.122		0.092	−0.024 to 0.205	0.119
		Controls (*n*=194)	0.107	−0.035 to 0.244	0.138		0.108	−0.034 to 0.247	0.134
		No secretagogue (*n*=43)	0.014	−0.288 to 0.313	0.930		−0.111	−0.408 to 0.208	0.493
		Any secretagogue (*n*=54)	−0.086	−0.346 to 0.186	0.535		−0.101	−0.366 to 0.180	0.479
								
CCL-3 (MIP-1α)	IL-1β	All subjects (*n*=291)	**0.151**	**0.037 to 0.261**	**0.010**		**0.156**	**0.041 to 0.267**	**0.008**
		Controls (*n*=194)	0.092	−0.050 to 0.229	0.203		0.092	−0.051 to 0.231	0.205
		No secretagogue (*n*=43)	**0.590**	**0.352 to 0.756**	**<0.001**		**0.618**	**0.380 to 0.780**	**<0.001**
		Any secretagogue (*n*=54)	0.093	−0.179 to 0.352	0.500		0.088	−0.192 to 0.355	0.536
								
CCL-3 (MIP-1α)	IL-1Ra	All subjects (*n*=291)	**0.232**	**0.120 to 0.338**	**<0.001**		**0.232**	**0.120 to 0.339**	**<0.001**
		Controls (*n*=194)	**0.223**	**0.085 to 0.353**	**0.002**		**0.215**	**0.076 to 0.347**	**0.003**
		No secretagogue (*n*=43)	**0.538**	**0.283 to 0.722**	**<0.001**		**0.553**	**0.291 to 0.737**	**<0.001**
		Any secretagogue (*n*=54)	0.247	−0.022 to 0.483	0.069		**0.278**	**0.003 to 0.514**	**0.046**
								
CCL-3 (MIP-1α)	TNF-α	All subjects (*n*=291)	**0.163**	**0.049 to 0.273**	**0.005**		**0.170**	**0.055 to 0.280**	**0.004**
		Controls (*n*=194)	0.112	−0.030 to 0.249	0.120		0.110	−0.033 to 0.248	0.129
		No secretagogue (*n*=43)	**0.570**	**0.325 to 0.743**	**<0.001**		**0.625**	**0.389 to 0.784**	**<0.001**
		Any secretagogue (*n*=54)	**0.313**	**0.049 to 0.536**	**0.020**		**0.301**	**0.028 to 0.533**	**0.030**
								
CCL-3 (MIP-1α)	IL-6	All subjects (*n*=291)	0.106	−0.009 to 0.219	0.070		0.110	−0.006 to 0.223	0.062
		Controls (*n*=194)	0.092	−0.050 to 0.230	0.202		0.101	−0.042 to 0.239	0.165
		No secretagogue (*n*=43)	0.296	−0.005 to 0.548	0.051		**0.309**	−**0.002 to 0.566**	**0.049**
		Any secretagogue (*n*=54)	0.132	−0.141 to 0.386	0.340		0.142	−0.139 to 0.401	0.319
								
CCL-4 (MIP-1β)	CCL-5 (RANTES)	All subjects (*n*=291)	−0.009	−0.124 to 0.106	0.872		−0.008	−0.123 to 0.108	0.894
		Controls (*n*=194)	−0.039	−0.179 to 0.102	0.588		−0.038	−0.179 to 0.105	0.601
		No secretagogue (*n*=43)	0.185	−0.122 to 0.460	0.230		0.205	−0.114 to 0.485	0.201
		Any secretagogue (*n*=54)	−0.037	−0.302 to 0.233	0.789		−0.055	−0.326 to 0.224	0.700
								
CCL-4 (MIP-1β)	IL-1β	All subjects (*n*=291)	**0.574**	**0.491 to 0.646**	**<0.001**		**0.574**	**0.491 to 0.647**	**<0.001**
		Controls (*n*=194)	**0.217**	**0.079 to 0.347**	**0.002**		**0.217**	**0.078 to 0.348**	**0.002**
		No secretagogue (*n*=43)	**0.920**	**0.855 to 0.956**	**<0.001**		**0.920**	**0.852 to 0.957**	**<0.001**
		Any secretagogue (*n*=54)	0.018	−0.251 to 0.285	0.895		0.037	−0.241 to 0.310	0.795
								
CCL-4 (MIP-1β)	IL-1Ra	All subjects (*n*=291)	**0.836**	**0.798 to 0.868**	**<0.001**		**0.836**	**0.798 to 0.868**	**<0.001**
		Controls (*n*=194)	**0.875**	**0.838 to 0.905**	**<0.001**		**0.875**	**0.838 to 0.905**	**<0.001**
		No secretagogue (*n*=43)	**0.861**	**0.757 to 0.923**	**<0.001**		**0.862**	**0.752 to 0.925**	**<0.001**
		Any secretagogue (*n*=54)	**0.365**	**0.107 to 0.576**	**0.006**		**0.420**	**0.163 to 0.623**	**0.002**
								
CCL-4 (MIP-1β)	TNF-α	All subjects (*n*=291)	**0.438**	**0.340 to 0.527**	**<0.001**		**0.446**	**0.349 to 0.534**	**<0.001**
		Controls (*n*=194)	**0.421**	**0.298 to 0.531**	**<0.001**		**0.430**	**0.307 to 0.539**	**<0.001**
		No secretagogue (*n*=43)	**0.450**	**0.173 to 0.661**	**0.002**		**0.501**	**0.224 to 0.703**	**<0.001**
		Any secretagogue (*n*=54)	0.067	−0.238 to 0.360	0.667		**0.376**	**0.112 to 0.591**	**0.006**
								
CCL-4 (MIP-1β)	IL-6	All subjects (*n*=291)	**0.334**	**0.228 to 0.433**	**<0.001**		**0.336**	**0.230 to 0.435**	**<0.001**
		Controls (*n*=194)	**0.317**	**0.184 to 0.438**	**<0.001**		**0.322**	**0.188 to 0.443**	**<0.001**
		No secretagogue (*n*=43)	**0.680**	**0.477 to 0.814**	**<0.001**		**0.693**	**0.486 to 0.826**	**<0.001**
		Any secretagogue (*n*=54)	0.190	−0.082 to 0.436	0.165		0.217	−0.063 to 0.464	0.123
								
CCL-5 (RANTES)	IL-1β	All subjects (*n*=291)	0.037	−0.079 to 0.151	0.535		0.040	−0.076 to 0.155	0.500
		Controls (*n*=194)	0.081	−0.060 to 0.220	0.258		0.088	−0.055 to 0.227	0.225
		No secretagogue (*n*=43)	0.056	−0.249 to 0.350	0.722		0.065	−0.251 to 0.369	0.687
		Any secretagogue (*n*=54)	0.095	−0.178 to 0.353	0.494		0.098	−0.182 to 0.364	0.489
								
CCL-5 (RANTES)	IL-1Ra	All subjects (*n*=291)	0.008	−0.107 to 0.123	0.895		0.008	−0.107 to 0.124	0.888
		Controls (*n*=194)	0.011	−0.130 to 0.152	0.874		0.013	−0.129 to 0.155	0.857
		No secretagogue (*n*=43)	0.052	−0.252 to 0.347	0.739		0.040	−0.275 to 0.347	0.804
		Any secretagogue (*n*=54)	−0.093	−0.352 to 0.179	0.502		−0.123	−0.386 to 0.158	0.386
								
CCL-5 (RANTES)	TNF-α	All subjects (*n*=291)	−0.064	−0.178 to 0.051	0.274		−0.047	−0.162 to 0.069	0.422
		Controls (*n*=194)	−**0.146**	−**0.281 to** −**0.005**	**0.042**		−**0.143**	−**0.279 to** −**0.001**	**0.048**
		No secretagogue (*n*=43)	0.067	−0.238 to 0.360	0.667		0.089	−0.229 to 0.390	0.582
		Any secretagogue (*n*=54)	0.104	−0.168 to 0.362	0.451		0.103	−0.178 to 0.368	0.470
								
CCL-5 (RANTES)	IL-6	All subjects (*n*=291)	0.051	−0.065 to 0.165	0.388		0.047	−0.069 to 0.161	0.430
		Controls (*n*=194)	0.043	−0.098 to 0.183	0.546		0.042	−0.100 to 0.183	0.562
		No secretagogue (*n*=43)	0.038	−0.266 to 0.334	0.810		0.052	−0.264 to 0.358	0.749
		Any secretagogue (*n*=54)	0.126	−0.147 to 0.381	0.362		0.166	−0.115 to 0.422	0.242

Significant correlations are displayed in bolded text. The differences that are only significant in either adjusted or unadjusted correlations are further denoted by an outline. Chemokine ligand 2, CCL-2 (monocyte chemoattractant protein 1, MCP-1); chemokine ligand 3, CCL-3 (macrophage inflammatory protein 1α, MIP-1α); chemokine ligand 4, CCL-4 (macrophage inflammatory protein 1β, MIP-1β); chemokine ligand 5, CCL-5 (regulated on activation normal T cell expressed and secreted, RANTES); tumor necrosis factor α, TNF-α; interleukin 1β, IL-1β; interleukin 1β receptor antagonist, IL-1Ra; interleukin 6, IL-6.
