# Supplementary material for: Insulin secretagogue use and circulating inflammatory C–C chemokine levels in breast cancer patients
Source: Data Brief. 2017 Feb 16;11:391–402. doi: 10.1016/j.dib.2017.02.031 (PMC5331147; doi:10.1016/j.dib.2017.02.031)
Supplement: Supplementary file 1 — Supplementary material [file mmc1.pdf]

AUTHOR DECLARATION

We wish to confirm that there are no known conflicts of interest associated with the publication **"Insulin secretagogue use and circulating inflammatory cytokine levels in breast cancer patients"** (submitted to Elsevier's Data-in-Brief) and there has been no significant financial support for this work that could have influenced its outcome.

We confirm that the manuscript has been read and approved by all named authors and that there are no other persons who satisfied the criteria for authorship but are not listed. We further confirm that the order of authors listed in the manuscript has been approved by all of us.

We confirm that we have given due consideration to the protection of intellectual property associated with this work and that there are no impediments to publication, including the timing of publication, with respect to intellectual property. In so doing we confirm that we have followed the regulations of our institutions concerning intellectual property.

We further confirm that any aspect of the work covered in this manuscript that has involved human patients has been conducted with the ethical approval of all relevant bodies and that such approvals are acknowledged within the manuscript.

We understand that the Corresponding Author – Dr Alice C. Ceacareanu - is the sole contact for the Editorial process (including Editorial Manager and direct communications with the office). She is responsible for communicating with other authors about progress, submissions of revisions and final approval proofs. We confirm that we have provided a current, correct email address which is accessible by Dr Alice C. Ceacareanu (Corresponding Author) and which has been configured to accept email from ACC36@BUFFALO.EDU.

Zachary A. P. Wintrob (zwintrob@gmail.com) [signature] Date 11/11/16

Jeffrey P. Hammel (jhammel@icpd.com) Jeffrey Hammel Date 11/9/16

George K. Nimako (gknimako@gmail.com) [signature] Date 11/02/16

Zahra S. Fayazi (zsfayazi@buffalo.edu) Zahra Fayazi Date 11/10/16

Dan P. Gaile (dpgaile@buffalo.edu) [signature] Date 11/11/16

Alan Forrest (systat10@email.unc.edu) [signature] Date 11/07/16

Alice C. Ceacareanu (acc36@buffalo.edu) [signature] Date 11/10/16
